# Supplementary material for: Patterns of Intron Gain and Loss in Fungi
Source: PLoS Biol. 2004 Nov 30;2(12):e422. doi: 10.1371/journal.pbio.0020422 (PMC532390; doi:10.1371/journal.pbio.0020422)
Supplement: Table S1 — Also available at http://genes.mit.edu/NielsenEtAl/. (4.3 MB ZIP). [file pbio.0020422.st001.zip › NielsenEtAl/html/478.html]

AN1257.1.NCU00244.1.MG04092.1.FG10282.1


```
 CLUSTAL W (1.82) Multiple Sequence Alignments - Introns Inserted


Sequence 1: MG04092.1	318 aa
Sequence 2: FG10282.1	322 aa
Sequence 3: AN1257.1	332 aa
Sequence 4: NCU00244.1	311 aa
Alignment Length: 357 aa
Number Identitical Residues: 78 aa
Alignment Score (without introns) 5506


MG04092.1 	MAD---GKRAVDSS--RV1WTTLITNLDYLPGLLTLDHALRKHKSKYPLVALYTDTFPES
NCU00244.1	--------MAVDYPTNMI~WSTLVTKRAYLGGALVLNHSLKKVGSRYQLKIMVTREAQAD
FG10282.1 	MTEYEPRKRVVDSP--KV1WTTLITNLSYLPGLLTLDHSLRVAKSQYPLVALYTDTFPPE
AN1257.1  	MSGNISTNSNPNPNPKKV1WTTLITNSSYIPGLLTLEYSLRRCESKYPFVVLYTDSFPIS
          	 :   . .   :  .. : *:**:*:  *: * *.*:::*:   *:* :  : *     .

MG04092.1 	GR--TALASRCIAAQRIEYLLPTKSSRDYSEDPRFYDCWSKLTPFSLEQYD~RVVQLDSD
NCU00244.1	KEFMAVFAAAGIPTIVIETIEPARQ------GKVNKAFWQKLAPWAMTEYE0RIVLLDSD
FG10282.1 	GH--AALRARGIPTQHIPYLLPTKG-KDYSNDPRFYDCWSKLTPFSLTEYD~RVVQLDSD
AN1257.1  	GH--AALDARGIAKKHVPYLLPSIP-KDYTNDVRFYDCWSKLTPFSLTEYE~RVVQLDSD
          	 .  :.: :  *.   :  : *:    . :..      *.**:*::: :*: *:* ****

MG04092.1 	MLVLQNMDELMEMELDAPEVSAAGGPD-SKRVFAASYACVCNPLKRAHYPKDW~VPENCA
NCU00244.1	QVILQNIDHLMTLHLP-------------EGHIACSHACTCNPRKLAHYPKDW2VPQNCP
FG10282.1 	MLVLRNMDELMDLELDAPSIAETGDKTISKRVFAAGHACVCNPLKKPHYPKDW~VKENCA
AN1257.1  	MLILRNMDELMDLQLDGPEMKGE-----GSRVFGAAHACVCNPLKKPHYPPNW2VPSNCV
          	 ::*:*:*.** :.* ...         ..  :...:**.*** * .*** :* * .** 

MG04092.1 	FTTQHGEPDKAQVEGADPAKVPLPILNGGLQVVNPSRVLFQQIVEHMENN-AAS-LDFAD
NCU00244.1	FTSADQHT-GSPAPITPSSPRTHHLLNSGTVVLTPSKPQFDALLDAINTHPDVPHMVFPD
FG10282.1 	FTSQHSTPDIAQTEAADPSVGPLGFMNGGLQVVNPSQGLYAQIVAHMEAD-AVN-MDFAD
AN1257.1  	YTDQHSHPELAS-HIAPPASAALGIPNGGLQVVNPSLEIYNKIIAQLGSA-ATSSYDFAD
          	:*  .  .. :    : .:  .  : *.*  *:.**   :  ::  :     .    *.*

MG04092.1 	QSLLSELYKDRWVALPYVYNALKTLRWPGVHDAIWRDDKVKNVHYILTPNPWDQLDAE--
NCU00244.1	QDILAIVYRGKWKPLPYVYNALKPMR--DCHASLWRDEDVKILHYILN-KPWESRDFD--
FG10282.1 	QSLLSDLYRERWVPLPYIYNALKTMRWEGVHNTIWRDESVKNIHYILSPKPWDEINDK--
AN1257.1  	QSLLGDLFAGRWVALPYVYNALKTMRWRGVHDVIWKDAEVKNVHYILSPKPWEEDPEGRD
          	*.:*. ::  :* .***:*****.:*  . *  :*:* .** :****..:**:.     .

MG04092.1 	------------GNTKSEEETMGWWADVNKERR-------EGEKAKGIN-DGF-------
NCU00244.1	-------------ENDKVESTHRLWWGVWEEVEREWTGGEAGEEKRRLYDSVLRSVVAQA
FG10282.1 	------------GEWTGKDESHKWWVDVNRMRK-------EAEKEKGIPDDGF-------
AN1257.1  	QDQEQDGVEIRVGNSDTKDPLHDLWWKVNRERL-------REEKRRGIN-DRF-------
          	....... .   .:    :     *  * .            *: : :  . :       

MG04092.1 	
FG10282.1 	
AN1257.1  	
NCU00244.1	
          	
```
